# Microbiota–Gut–Brain Axis in Alzheimer’s Disease: Linking Oxidative Stress, Mitochondrial Dysfunction and Amyloid Pathology—A Systematic Review

**DOI:** 10.3390/biomedicines14040860

**Published:** 2026-04-09

**Authors:** Shah Rezlan Shajahan, Nurhidayah Hamid, Blaire Okunsai, Norshafarina Shari, Muhammad Danial Che Ramli

**Affiliations:** 1 School of Graduate Studies, Postgraduate Centre, Management and Science University, Shah Alam 40100, Selangor, Malaysia; shahrezlan@gmail.com (S.R.S.); norshafarina@msu.edu.my (N.S.); 2 Graduate School of Management, Postgraduate Centre, Management and Science University, Shah Alam 40100, Selangor, Malaysia; nurhidayah857@gmail.com; 3 Department of Diagnostic and Allied Health Science, Faculty Health and Life Sciences, Management and Science University, Shah Alam 40100, Selangor, Malaysia; hobi66blaire@gmail.com

**Keywords:** Alzheimer’s disease, gut microbiota, oxidative stress, mitochondrial dysfunction, neuroinflammation, probiotics, microbiota gut-brain axis, biomarkers

## Abstract

**Background:** Alzheimer’s disease (AD) is a multifactorial neurodegenerative disorder characterized by amyloid-β aggregation, tau hyperphosphorylation, oxidative stress, and mitochondrial dysfunction. Emerging evidence indicates that the gut microbiota plays a critical role in modulating neuroinflammatory, and metabolic pathways involved in AD pathogenesis through the microbiota-gut-brain axis. **Objective:** This systematic review aims to comprehensively evaluate the role of the microbiota-gut-brain axis in Alzheimer’s disease, with a particular focus on its mechanistic links to oxidative stress, mitochondrial dysfunction, and amyloid pathology, as well as its therapeutic potential. **Methodology:** A comprehensive literature search was conducted using PubMed, Scopus, and Web of Science databases, focusing on studies evaluating gut microbiota composition, metabolomic changes, oxidative stress markers, mitochondrial activity, and therapeutic interventions in AD models and patients. **Results:** Altered gut microbial composition in AD is associated with increased pro-inflammatory taxa (*Escherichia-Shigella*, *Bacteroides*) and depletion of short-chain fatty acid (SCFA) producing bacteria (*Faecalibacterium*, *Roseburia*). Dysbiosis contributes to systemic inflammation, disrupted intestinal permeability, and microglial activation, leading to oxidative damage and mitochondrial impairment in neurons. Preclinical and clinical studies indicate that probiotics, prebiotics, and fecal microbiota transplantation can restore redox balance, reduce neuroinflammation, and improve cognitive outcomes. Multi-omics and AI-based models are emerging as tools for identifying microbiome-derived biomarkers for early AD detection. **Conclusion:** The gut microbiota-mitochondria-oxidative stress axis represents a promising therapeutic target in Alzheimer’s disease. Future research should focus on longitudinal human studies, standardized microbial profiling, and personalized microbiome-based interventions to translate these mechanistic insights into clinical benefit.

## 1. Introduction

Alzheimer’s disease (AD) is a progressive, irreversible neurodegenerative disorder that currently affects over 55 million people worldwide, a number projected to triple by 2050 due to population aging [1]. Clinically, AD is characterized by a gradual decline in cognitive functions, including memory, executive ability, and language, eventually leading to total dependency and death [2]. Pathologically, AD is marked by hallmark features such as extracellular accumulation of amyloid-β (Aβ) plaques, intracellular neurofibrillary tangles (NFTs) formed by hyperphosphorylated tau protein, synaptic dysfunction, and extensive neuronal loss, particularly in the hippocampus and cortex [3,4,5]. Chronic neuroinflammation, oxidative stress, and mitochondrial dysfunction further exacerbate neuronal injury and accelerate disease progression.

Oxidative stress, resulting from the imbalance between reactive oxygen species (ROS) and antioxidant defences, accelerates lipid peroxidation, DNA damage, and protein misfolding, all of which exacerbate neurodegeneration [6]. Mitochondrial dysfunction further compounds these effects by impairing ATP production, disrupting calcium homeostasis, and reducing neuronal resilience to metabolic stress [7]. Simultaneously, amyloidogenic processes are aggravated under these conditions, with evidence suggesting that oxidative and mitochondrial dysfunction act as upstream modulators of Aβ accumulation and tau hyperphosphorylation [8]. The intertwined nature of these pathways underscores the need for therapeutic strategies that target AD beyond amyloid- and tau-centric approaches, which have repeatedly failed in late-stage clinical trials.

In this context, the gut-brain axis has emerged as a compelling modulator of neurodegenerative processes. This bidirectional communication system links the gastrointestinal tract and central nervous system through neuronal, endocrine, immune, and metabolic pathways [7,9]. Central to this axis is the gut microbiota, a complex microbial ecosystem that regulates host immunity, energy metabolism, and redox balance. Dysbiosis, a disruption in microbial composition and function, has been increasingly associated with neurodegenerative diseases, including AD [9,10]. Recent studies reveal that altered gut microbial communities contribute to enhanced oxidative stress, mitochondrial impairment, and amyloid deposition through metabolites such as lipopolysaccharides (LPS), short-chain fatty acids (SCFAs), bile acids, and tryptophan catabolites [7,11].

The integration of microbiota research with AD pathophysiology offers a transformative perspective: gut dysbiosis may not only exacerbate oxidative and mitochondrial damage but also amplify amyloidogenic cascades, thus accelerating cognitive decline. Therapeutically, targeting the microbiome through probiotics, prebiotics, dietary polyphenols, or faecal microbiota transplantation (FMT) has demonstrated promising results in reducing oxidative burden and modulating amyloid pathology in preclinical and early clinical studies [12,13].

Importantly, gut microbiota modulation represents a uniquely promising therapeutic axis in Alzheimer’s disease because it targets upstream, systemic, and modifiable drivers of neurodegeneration, rather than end-stage neuropathology alone. Unlike amyloid or tau deposition, which emerge after extensive neuronal damage, gut dysbiosis develops early and dynamically interacts with oxidative stress, mitochondrial dysfunction, immune activation, and metabolic imbalance throughout disease progression. Microbiota-targeted interventions therefore offer a multi-level strategy capable of simultaneously reducing oxidative burden, restoring mitochondrial efficiency, attenuating neuroinflammation, and indirectly modulating amyloidogenic pathways [9,11,13]. Moreover, the gut microbiome is highly responsive to non-invasive interventions, including diet, probiotics, prebiotics, and microbiota-derived metabolites, making it particularly amenable to long-term prevention and precision therapy. This positions microbiota modulation as a translationally feasible and biologically integrative approach that complements, rather than competing with, conventional amyloid and tau-focused strategies in AD.

This systematic review aims to comprehensively evaluate the role of the microbiota-gut-brain axis in Alzheimer’s disease, with a particular focus on its mechanistic links to oxidative stress, mitochondrial dysfunction, and amyloid pathology. Specifically, this review synthesizes current evidence to elucidate how gut microbiota dysbiosis contributes to key neurodegenerative processes and explores its potential as a therapeutic target for modulating disease progression.

### Familial and Sporadic Alzheimer’s Disease: Distinct Aetiologies, Shared Pathways

Alzheimer’s disease (AD) comprises two major etiological forms: familial (early-onset) AD (FAD) and sporadic (late-onset) AD (SAD). Familial AD accounts for less than 5% of total cases and is typically caused by autosomal dominant mutations in genes such as APP, PSEN1, and PSEN2, which directly increase amyloid-β (Aβ) production or alter its aggregation dynamics [3,14]. In contrast, sporadic AD represents over 95% of cases and arises from a complex interplay of aging, genetic susceptibility most notably the APOE ε4 allele metabolic dysfunction, vascular factors, environmental exposures, and systemic inflammation [15,16].

While FAD is primarily driven by deterministic genetic mutations leading to early and aggressive amyloid pathology, SAD develops gradually through multifactorial mechanisms involving oxidative stress, mitochondrial dysfunction, immune dysregulation, and metabolic imbalance [6,17]. Importantly, many of these systemic contributors are modifiable and interact bidirectionally with peripheral organs, including the gastrointestinal tract, thereby influencing neuroinflammatory and metabolic signalling pathways relevant to AD progression [18,19].

The microbiota-gut-brain axis is therefore particularly relevant in the context of sporadic AD. Unlike monogenic familial AD, sporadic AD reflects cumulative systemic stressors and environmental influences that shape redox homeostasis, mitochondrial resilience, and inflammatory tone [7,20]. Gut dysbiosis may amplify these systemic vulnerabilities by promoting endotoxemia, oxidative stress, metabolic dysfunction, and neuroinflammation processes that converge on amyloid and tau pathology [11,18].

Although microbiome alterations have also been observed in transgenic models of familial AD, microbiota-targeted interventions are most conceptually aligned with sporadic AD, where modifiable peripheral drivers substantially influence disease trajectory [21,22]. Throughout this review, we therefore interpret microbiome-related mechanisms primarily within the framework of sporadic AD while acknowledging shared downstream neuropathological pathways between familial and sporadic forms.

## 2. Methods

### 2.1. Search Strategy

A comprehensive literature search was conducted using the PubMed, Scopus, and Web of Science databases to identify relevant studies published between 2016 and 2026. The search strategy combined keywords and Medical Subject Headings (MeSH), including: “Alzheimer’s disease”, “gut microbiota”, “microbiota-gut-brain axis”, “oxidative stress”, and “mitochondrial dysfunction”. Boolean operators (AND, OR) were applied to refine the search.

### 2.2. Eligibility Criteria

Studies were included if they:

(i) Investigated the relationship between gut microbiota and Alzheimer’s disease;
(ii) Examined oxidative stress, mitochondrial dysfunction, or amyloid pathology;
(iii) Were original research articles (clinical and preclinical studies);
(iv) Were published in English.

Studies were excluded if they:

(i) Were unrelated to Alzheimer’s disease or gut microbiota;
(ii) Lacked full-text availability;
(iii) Were conference abstracts, editorials, or duplicate publications.

### 2.3. Study Selection Process

A total of 450 records were identified through database searching. After removal of duplicates (*n* = 130), 320 records were screened based on titles and abstracts. Of these, 210 records were excluded due to irrelevance, incomplete data, or lack of accessibility. A total of 110 full-text articles were assessed for eligibility, resulting in 88 studies were included in the final qualitative synthesis.

**Figure 1:**
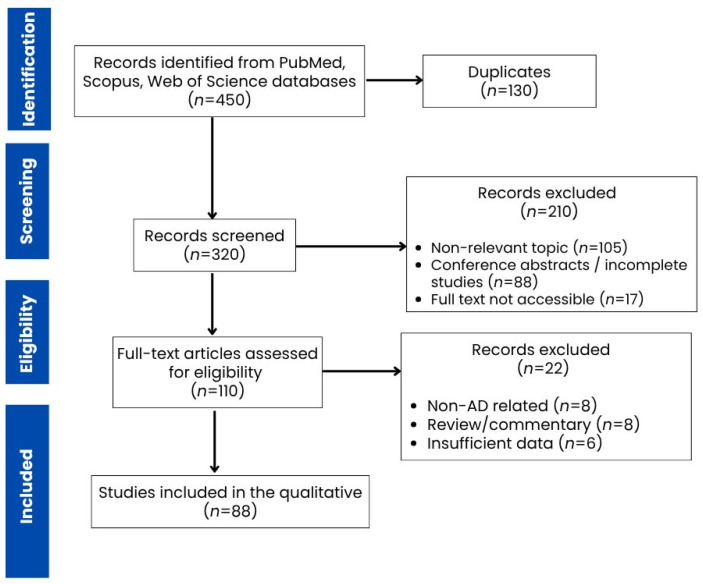
PRISMA flow diagram of the study selection process.

The screening process was conducted through title and abstract screening followed by full-text assessment. The study selection process is illustrated in the PRISMA flow diagram (Figure 1).

### 2.4. Data Extraction and Synthesis

Relevant data were extracted from included studies, including study design, population/model, microbiota composition, associated pathological mechanisms (oxidative stress, mitochondrial dysfunction, amyloid pathology), and key findings. A narrative synthesis approach was used to integrate findings across studies, focusing on mechanistic pathways and therapeutic implications. A formal risk of bias assessment was not conducted due to the narrative nature of the synthesis.

### 2.5. Reporting Standards

This review was conducted and reported in accordance with the Preferred Reporting Items for Systematic Reviews and Meta-Analyses (PRISMA) 2020 guidelines (Supplementary Material) [23]. The review protocol was registered with the Open Science Framework (OSF), (Registration DOI: https://doi.org/10.17605/OSF.IO/38E9D).

## 3. Alzheimer’s Disease Pathophysiology in Context

Alzheimer’s disease (AD) is a multifactorial neurodegenerative disorder in which classical neuropathology—extracellular amyloid-β (Aβ) plaques and intracellular neurofibrillary tangles (NFTs) of hyperphosphorylated tau—is coupled to progressive synaptic dysfunction and neuronal loss predominantly in hippocampal and cortical circuits [4]. Aβ and tau remain central to diagnostic frameworks and biomarker development, it is noteworthy that in some cases, post-mortem analyses reveal Aβ accumulation without preceding clinical signs of cognitive impairment, indicating that amyloid deposition alone is not always predictive of symptomatic disease [24,25]. Their relationship to clinical decline is therefore neither linear nor singular: tau burden tracks clinical severity more closely than amyloid burden, and both interact with inflammatory, metabolic, and vascular processes that determine trajectory and rate of decline [18].

Neuroinflammation and synaptic failure are essential components of AD pathophysiology. Microglial activation and a chronic pro-inflammatory milieu contribute to synaptic pruning, impaired plasticity, and perpetuation of neurodegenerative cascades [26]. Synaptic dysfunction, the earliest substrate for cognitive impairment arises from Aβ oligomer toxicity, tau-mediated cytoskeletal disruption, and dysfunction of cellular bioenergetics that together impair neurotransmission and network integrity [6,18].

Importantly, immune signalling in AD extends beyond the central nervous system and involves dynamic cross-talk between the gut, peripheral immune system, and brain. The gastrointestinal tract houses the largest immune compartment in the body, where gut-associated lymphoid tissue (GALT) continuously interacts with microbial antigens and metabolites. Under physiological conditions, commensal microbiota promote immune tolerance and maintain epithelial barrier integrity. However, dysbiosis may increase intestinal permeability, facilitating systemic translocation of microbial-derived components such as lipopolysaccharide (LPS) and bacterial amyloids. These molecules activate peripheral innate immune pathways through pattern recognition receptors, including Toll-like receptors (TLRs), driving sustained production of pro-inflammatory cytokines (e.g., IL-1β, IL-6, TNF-α) that amplify systemic inflammatory tone [27,28].

Peripheral inflammatory mediators can influence central immunity by compromising blood–brain barrier integrity and priming microglia toward a pro-inflammatory phenotype. Chronically activated microglia enhance oxidative stress, impair synaptic plasticity, and promote amyloidogenic processing and tau phosphorylation, thereby reinforcing neurodegenerative cascades. This gut-peripheral-brain immune axis provides a mechanistic framework linking microbiome alterations to canonical AD pathology and may be particularly relevant in sporadic AD, where cumulative metabolic and inflammatory stressors shape disease trajectory rather than deterministic genetic mutations alone.

Despite large investments in Aβ and tau-directed therapeutics, clinical outcomes have been modest and inconsistent. Large meta-analyses and systematic assessments of anti-Aβ monoclonal antibodies show evidence of robust target engagement (amyloid lowering) but only modest and often clinically marginal slowing of cognitive decline; benefits, when present, are small and accompanied by safety concerns such as ARIA (amyloid-related imaging abnormalities) [29]. Critical commentary and recent reviews emphasize that (1) amyloid removal may be necessary but not sufficient, (2) amyloid and tau are downstream manifestations in many patients, and (3) treatment timing, patient selection, and multimodal pathophysiology complicate translation of amyloid clearance into meaningful clinical benefit [15,29]. Consequently, the field increasingly recognizes that single-target strategies are unlikely to fully arrest disease progression, particularly in sporadic AD, where systemic, metabolic, and environmental factors significantly influence disease trajectory, in contrast to the deterministic genetic mutations driving familial AD.

A growing body of evidence therefore points to systemic contributors that converge on neuronal vulnerability: oxidative stress, mitochondrial dysfunction, metabolic insufficiency, vascular compromise, and peripheral immune activation [6,26]. Oxidative stress, generated by excessive reactive oxygen and nitrogen species relative to endogenous antioxidant capacity drives lipid peroxidation, protein oxidation, DNA damage, and post-translational modifications that potentiate Aβ aggregation and tau phosphorylation [17]. Mitochondrial dysfunction, including disrupted electron transport chain activity, impaired mitophagy, and disturbed calcium handling, undermines neuronal ATP supply and increases ROS output, creating a self-amplifying cycle of energy failure and oxidative injury [17,26]. Importantly, mitochondrial abnormalities are observed early in AD and correlate with synaptic loss and cognitive impairment, suggesting mitochondria are not merely bystanders but key effectors of neuronal vulnerability [26].

Direct mitochondria-microbiota communication provides an additional layer of mechanistic integration between peripheral dysbiosis and neuronal vulnerability. Given the evolutionary origin of mitochondria from ancestral α-proteobacteria, mitochondria retain structural and functional similarities to bacteria, including cardiolipin-containing membranes and circular DNA, rendering them highly responsive to microbial-derived metabolites and immune signals [20,30]. Short-chain fatty acids (SCFAs), secondary bile acids, tryptophan metabolites, and bacterial components such as lipopolysaccharide (LPS) can influence mitochondrial bioenergetics, redox balance, and dynamics (fission–fusion cycling). In intestinal epithelial cells, dysbiosis-associated metabolites alter mitochondrial oxidative phosphorylation and ROS generation, thereby compromising barrier integrity. Systemically, circulating microbial metabolites and inflammatory mediators may impair neuronal mitochondrial respiration, promote excessive ROS production, and disrupt mitophagy, creating conditions that favor amyloidogenic processing and tau pathology [31,32]. Thus, mitochondria function as central bioenergetic and immunometabolism sensors of microbial signals, linking gut dysbiosis directly to neuronal metabolic stress and neurodegenerative cascades.

Recent meta-analytic and mechanistic studies have also positioned the gut microbiota and the broader microbiota-gut-brain axis as modulators of these systemic pathways [18,33]. Human and animal studies reveal reproducible shifts in microbial composition and function in AD, with loss of SCFA-producing and anti-inflammatory taxa and enrichment of proinflammatory bacteria. Such dysbiosis can increase systemic endotoxin exposure (e.g., LPS), alter peripheral immunity, perturb bile acid and tryptophan metabolism, and shift redox balance all mechanisms that plausibly exacerbate oxidative stress, impair mitochondrial resilience, and promote amyloidogenic processing in the brain [18,33]. Thus, gut-derived signals may act upstream or in parallel with canonical CNS mechanisms to accelerate synaptic failure and neurodegeneration.

Taken together, contemporary evidence supports a systems view of AD: amyloid and tau remain central pathological markers, but disease initiation and progression are shaped by interacting systemic processes—oxidative stress, mitochondrial failure, inflammation, vascular dysfunction, and peripheral factors such as gut dysbiosis. This integrative perspective implies that effective disease modification will likely require combination or multi-targeted interventions that restore metabolic resilience, blunt oxidative injury, and modulate peripheral contributors, in addition to addressing protopathic aggregates [17,26,29]

## 4. Evidence of Gut Dysbiosis in Alzheimer’s Disease

### 4.1. Human Clinical Studies

**Figure 2:**
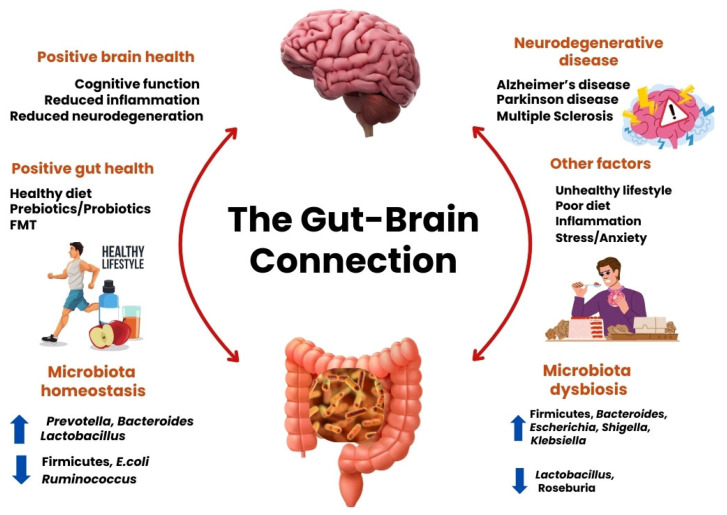
The gut-brain axis in health and neurodegeneration. Schematic illustration of the bidirectional communication between the gut microbiota and the brain. A healthy lifestyle and diet promote microbiota homeostasis, characterized by enrichment of beneficial taxa and increased production of neuroprotective microbial metabolites, thereby supporting redox balance, mitochondrial function, and positive mental health. In contrast, unhealthy lifestyle factors, poor diet, stress, inflammation, and antibiotic exposure drive gut dysbiosis, marked by expansion of pro-inflammatory pathobionts and depletion of short-chain fatty acid producing bacteria. Dysbiotic states contribute to systemic inflammation, oxidative stress, and neuroinflammatory signalling, increasing vulnerability to neurodegenerative disorders, including Alzheimer’s disease. This framework highlights the gut microbiota as a modifiable and therapeutically actionable axis in neurodegeneration. (Created in Canva Pro Version).

Mounting clinical evidence indicates that patients with Alzheimer’s disease (predominantly sporadic, late-onset cases in these cohorts) harbour a distinct gut microbial profile compared to cognitively healthy individuals. High-throughput 16S rRNA sequencing and shotgun metagenomics have consistently revealed reduced microbial diversity and shifts in key taxa. For example, AD cohorts exhibit depleted levels of anti-inflammatory and butyrate producing genera such as *Faecalibacterium*, *Roseburia*, and *Bifidobacterium* while showing enrichment in pro-inflammatory pathobionts such as *Escherichia/Shigella*, *Enterobacteriaceae*, and *Ruminococcus gnavus* [34,35]. Additional alterations have also been reported, including reductions in beneficial genera such as *Eubacterium* and *Prevotella*, alongside increased abundance of taxa linked to inflammation and metabolic dysregulation [36,37,38]. These microbial imbalances are strongly associated with elevated systemic inflammation and increased oxidative stress markers, both of which exacerbate neuronal injury and accelerate amyloid pathology [39]. Evidence from animal studies further supports these observations, as germ-free or antibiotic-treated AD mouse models demonstrate altered amyloid deposition and neuroinflammatory responses following manipulation of the gut microbiota, highlighting a mechanistic role of microbial dysbiosis in AD progression. These clinical patterns are consistent with a broader model of gut-brain axis dysfunction linking lifestyle, microbial composition, and neurodegeneration (Figure 2).

Interestingly, several studies have also reported that a subset of AD patients experience gastrointestinal (GI) symptoms, including constipation, altered bowel habits, and dyspepsia, which may correlate with gut microbiota alterations [40,41]. While the prevalence and severity of GI symptoms vary across cohorts, their presence supports a bidirectional gut-brain relationship and suggests that microbial dysbiosis may contribute not only to neurodegeneration but also to peripheral GI manifestations.

Recent metagenomic-metabolomic studies extend these findings by linking gut dysbiosis to circulating metabolites and amyloid burden. Patients with AD show altered levels of microbial-derived short-chain fatty acids (SCFAs), particularly a reduction in butyrate, which is known to promote mitochondrial efficiency and antioxidant defences [42]. Conversely, elevated lipopolysaccharides (LPS) from Gram-negative taxa have been detected in the plasma and even co-localized with amyloid plaques in post-mortem AD brains, implicating bacterial endotoxins in amyloid aggregation and microglial activation [43]. Furthermore, faecal microbiome signatures have been correlated with cerebrospinal fluid (CSF) biomarkers, including increased phosphorylated tau and reduced Aβ42, suggesting that gut microbial alterations may serve as non-invasive biomarkers of disease progression [7].

Collectively, these findings establish a clinical link between gut dysbiosis and systemic drivers of AD pathology. Table 1 summarizes the key microbial alterations reported in human studies, highlighting their associations with cognition, amyloid pathology, oxidative stress, and neuroinflammation.

### 4.2. Animal Model Findings

Animal studies provide mechanistic evidence that gut dysbiosis is not merely correlative but causally implicated in AD pathogenesis. Germ-free (GF) transgenic mouse models of AD demonstrate significantly reduced amyloid-β plaque deposition compared to conventionally colonized counterparts, suggesting that the presence of gut microbiota accelerates amyloid pathology [9]. Similarly, antibiotic-treated APP/PS1 mice show decreased neuroinflammation, lower oxidative stress levels, and attenuated plaque load, further highlighting the microbiota’s role in modulating neurodegeneration [48,50].

In addition to mechanistic findings, several animal studies have characterized specific alterations in gut microbial composition associated with AD pathology. Transgenic AD mouse models, including APP/PS1 and 5xFAD mice, frequently display reduced microbial diversity and shifts in bacterial taxa like those reported in human AD cohorts [38]. These changes include decreased abundance of beneficial SCFA-producing genera such as *Lactobacillus*, *Bifidobacterium*, and *Ruminococcaceae*, alongside increased levels of pro-inflammatory taxa including *Escherichia/Shigella*, *Helicobacteraceae*, and *Desulfovibrio*. Such microbial shifts are associated with enhanced neuroinflammation, impaired intestinal barrier integrity, and increased amyloid deposition in the brain, further supporting the causal contribution of gut dysbiosis to AD progression.

Microbiota transplantation experiments have provided the most compelling causal evidence. When fecal microbiota from AD patients are transplanted into germ-free or antibiotic-treated mice, recipients develop enhanced amyloid accumulation, mitochondrial dysfunction, and cognitive impairments, effectively recapitulating human AD phenotypes [51]. Conversely, colonization with microbiota from cognitively healthy donors confers neuroprotection, preserving synaptic function and reducing oxidative damage. These findings suggest that microbial composition directly shapes the brain’s redox balance and amyloidogenic environment.

Notably, microbial metabolites act as mediators in these processes. Reduced SCFAs and increased LPS in dysbiotic states impair mitochondrial energy metabolism, elevate ROS production, and enhance amyloid fibrillogenic [17,39]. Moreover, tryptophan catabolites and secondary bile acids derived from dysbiotic microbiota disrupt blood–brain barrier integrity and activate pro-inflammatory microglial pathways, accelerating neurodegeneration [29].

Together, human and animal studies converge to demonstrate that gut dysbiosis drives oxidative stress, mitochondrial dysfunction, and amyloid pathology establishing the microbiota-gut-brain axis as a pivotal contributor to AD progression. Table 1 also incorporates findings from animal models, consolidating the translational evidence that dysbiotic microbial shifts directly shape AD pathophysiology.

## 5. Mechanistic Intersections: Gut Microbiota and AD Pathways

To integrate these complex interactions, Table 2 summarizes the major mechanistic pathways through which gut dysbiosis intersects with core Alzheimer’s disease pathologies, highlighting key microbial alterations, downstream molecular consequences, and representative evidence.

### 5.1. Oxidative Stress

**Figure 3:**
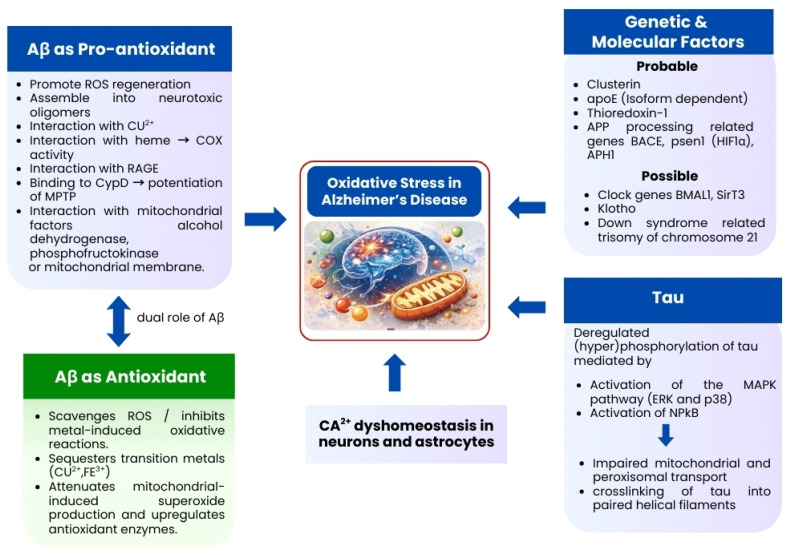
Oxidative stress-centered mechanisms driving neuronal damage in Alzheimer’s disease. Schematic representation of oxidative stress as a central pathological hub in Alzheimer’s disease (AD). Amyloid-β (Aβ) can exert pro-oxidant or antioxidant effects depending on aggregation state, redox kinetics, and disease stage, contributing directly and indirectly to reactive oxygen species (ROS) generation, mitochondrial dysfunction, and calcium (Ca^2+^) dyshomeostasis. Interactions of Aβ with metal ions, mitochondrial proteins, and receptor for advanced glycation end products (RAGE) further amplify oxidative injury. Genetic and molecular factors, including APOE isoforms, APP processing genes, and redox-regulatory pathways, modulate susceptibility to oxidative stress. Concurrently, oxidative stress promotes tau hyperphosphorylation through activation of stress-responsive kinases, impairing mitochondrial transport and cytoskeletal integrity. These converging pathways ultimately lead to synaptic dysfunction, neuronal damage, and cognitive decline, highlighting oxidative stress as a key mechanistic intersection linking upstream triggers, including gut microbiota-derived factors, to AD progression (Created in Canva Pro version).

Oxidative stress is one of the earliest and most persistent features of Alzheimer’s disease, and accumulating evidence highlights the gut microbiota as a significant upstream modulator of redox homeostasis. Dysbiosis promotes reactive oxygen species (ROS) overproduction by releasing pro-oxidant microbial metabolites, such as lipopolysaccharides (LPS) and trimethylamine-N-oxide (TMAO), which activate NADPH oxidase and mitochondrial ROS pathways [53]. In addition, mitochondrial dysfunction itself represents a major intracellular source of reactive oxygen species, as impaired electron transport chain activity leads to electron leakage and excessive ROS generation, thereby further amplifying oxidative stress in neuronal cells. Simultaneously, reduced levels of beneficial short-chain fatty acids (SCFAs), such as butyrate, weaken endogenous antioxidant defences by downregulating nuclear factor erythroid 2-related factor 2 (Nrf2) signalling [54]. Clinical studies have reported that patients with AD exhibit increased systemic markers of oxidative stress (e.g., malondialdehyde, 8-OHdG) correlated with altered microbial taxa, suggesting that microbial dysregulation directly exacerbates oxidative injury [6,52]. These findings support a model in which gut dysbiosis sustains a chronic pro-oxidative environment that accelerates neuronal injury and amyloidogenic processes. These interconnected oxidative pathways converge on mitochondrial dysfunction, amyloid and tau pathology, and neuronal injury, as summarized in Figure 3.

### 5.2. Mitochondrial Dysfunction

**Figure 4:**
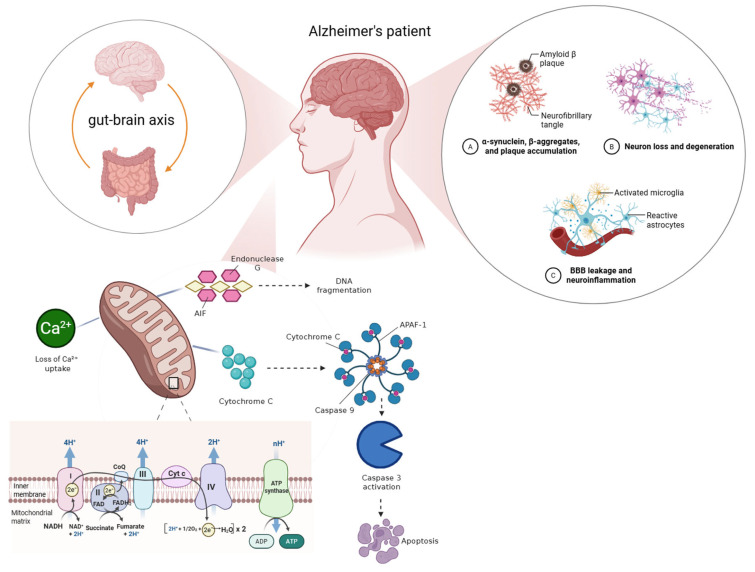
Mitochondrial dysfunction as a central mediator linking gut-brain axis alterations to Alzheimer’s disease pathology. Schematic overview of mitochondrial dysfunction in Alzheimer’s disease (AD) integrating gut-brain axis signalling, metabolic impairment, and neurodegeneration. Gut microbiota–derived factors influence brain energy metabolism through modulation of glucose utilization, insulin signalling, and mitochondrial tricarboxylic acid (TCA) cycle activity, leading to reduced ATP production and calcium (Ca^2+^) dyshomeostasis. Impaired mitochondrial function amplifies oxidative stress and promotes amyloid-β aggregation, neurofibrillary tangle formation, neuroinflammation, and blood–brain barrier (BBB) disruption. These interconnected processes culminate in synaptic failure, neuronal loss, and cognitive decline, positioning mitochondrial dysfunction as a critical nexus through which gut dysbiosis contributes to AD progression (Created with BioRender.com).

Mitochondrial deficits are central to neuronal degeneration in AD, with gut-derived metabolites emerging as potent disruptors of mitochondrial homeostasis. In addition, excessive oxidative stress can directly damage mitochondrial DNA, proteins, and membrane lipids, impairing electron transport chain activity and further exacerbating mitochondrial dysfunction in neuronal cells. LPS and bacterial amyloids penetrate systemic circulation, inducing mitochondrial permeability transition, calcium overload, and ATP depletion in neuronal cells [20]. Concurrently, dysbiosis reduces production of neuroprotective SCFAs and indole derivatives that normally sustain mitochondrial biogenesis and antioxidant enzyme activity [30]. Recent animal studies demonstrated that faecal microbiota transplantation (FMT) from AD patients into germ-free mice resulted in impaired oxidative phosphorylation and reduced expression of mitochondrial complex enzymes, linking microbial imbalance to bioenergetic deficits [55]. Importantly, mitochondrial dysfunction not only compromises energy metabolism but also amplifies ROS generation and facilitates Aβ aggregation, placing it at the nexus of gut microbiota–mediated neurodegeneration. The central role of mitochondrial dysfunction in linking gut-brain axis alterations to amyloid accumulation, neuroinflammation, and neuronal loss is illustrated in Figure 4.

### 5.3. Amyloid Pathology

The pathological aggregation of amyloid-β (Aβ) and hyperphosphorylation of tau remain defining hallmarks of AD. Gut dysbiosis appears to accelerate these processes via multiple routes. Pro-inflammatory taxa, such as *Escherichia/Shigella*, release LPS and bacterial amyloids (e.g., curli fibers) that cross-seed with host Aβ and promote prion-like misfolding [58]. Additionally, microbial metabolites such as secondary bile acids disrupt protein homeostasis, fostering Aβ oligomerization and tau phosphorylation [16]. Germ-free and antibiotic-treated mouse models consistently show reduced amyloid plaque burden, while recolonization with AD-derived microbiota restores Aβ pathology, providing strong causal evidence [58]. Collectively, these findings suggest that microbial-derived signals act as accelerators of amyloidogenic cascades, linking peripheral dysbiosis to central misfolding events in AD.

### 5.4. Neuroinflammation and Blood–Brain Barrier (BBB) Integrity

Neuroinflammation is increasingly recognized as a primary driver of AD progression, with gut-derived immune and metabolic signals acting as powerful modulators. Dysbiotic microbiota enhance systemic inflammation through translocation of LPS, which triggers Toll-like receptor 4 (TLR4)-mediated microglial activation and pro-inflammatory cytokine release [14,59]. Simultaneously, depletion of SCFA-producing bacteria reduces anti-inflammatory signalling, exacerbating chronic neuroinflammation [60]. Disruption of BBB integrity represents another key link: microbial metabolites such as LPS and p-cresol weaken tight junction proteins, allowing peripheral toxins and cytokines to penetrate the brain [41]. Additionally, gut-brain communication via the vagus nerve has been shown to mediate inflammatory responses, with altered vagal signalling in dysbiotic states promoting microglial overactivation [57]. Together, these mechanisms demonstrate how the microbiota acts as a systemic driver of neuroinflammation and barrier dysfunction, further amplifying oxidative and amyloidogenic cascades.

## 6. Microbiota-Based Therapeutic Interventions

**Figure 5:**
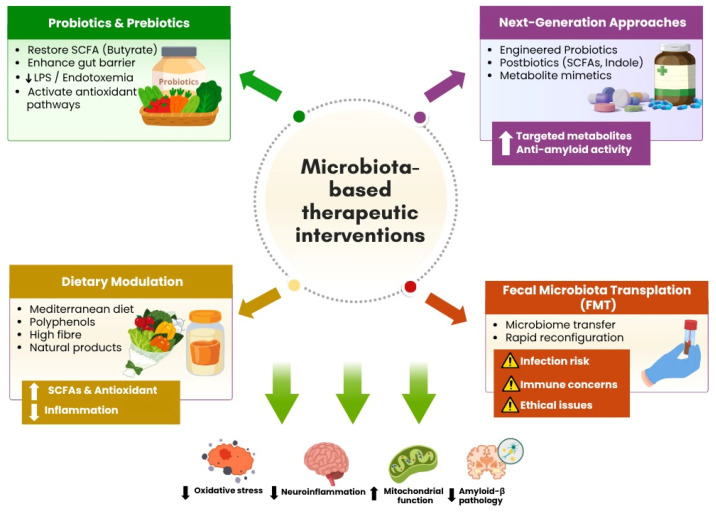
Microbiota-based therapeutic interventions in Alzheimer’s disease. Overview of major strategies targeting the gut-brain axis to modulate microbial composition and metabolic activity in Alzheimer’s disease. Key approaches include probiotics and prebiotics that promote beneficial microbial taxa and short-chain fatty acid production, dietary modulation that enhances antioxidant capacity and reduces pro-inflammatory signaling, fecal microbiota transplantation (FMT) for microbiome restoration, and emerging next-generation approaches such as engineered probiotics and targeted microbial metabolites. Collectively, these strategies aim to restore microbial balance, reduce neuroinflammation and oxidative stress, and support cognitive function through microbiota-mediated mechanisms. (Created in Canva Pro Version).

Therapeutic modulation of the gut microbiota has emerged as a multi-target strategy particularly relevant to sporadic AD, where systemic metabolic and inflammatory contributors play a central pathogenic role. Table 3 and Figure 5 summarize the principal microbiota-based interventions, their mechanistic rationale, current preclinical and clinical evidence, and key translational considerations, which are discussed in detail in the following sections.

### 6.1. Probiotics & Prebiotics—Antioxidant and Anti-Amyloid Effects

Rationale: Probiotics (live beneficial microbes) and prebiotics (non-digestible substrates that promote beneficial microbes) act via multiple mechanisms relevant to AD: restoring SCFA production (notably butyrate), strengthening intestinal barrier integrity, reducing systemic LPS/endotoxemia, upregulating host antioxidant pathways (e.g., Nrf2/GSH), and modulating peripheral and central immune responses [51,54].

Preclinical evidence: In AD rodent models, selected probiotic strains (e.g., *Lactobacillus rhamnosus*, *Bifidobacterium longum*) improved spatial memory, decreased hippocampal ROS, upregulated mitochondrial biogenesis markers, and reduced Aβ burden [65,66]. A recent comprehensive review summarizing probiotic intervention studies in transgenic AD rodent models further support the therapeutic potential of microbiome modulation in improving cognitive outcomes and reducing neuroinflammation [67]. Mechanistic studies indicate that butyrate and other SCFAs mediate many of these benefits by enhancing mitochondrial efficiency and activating antioxidant gene programs [60].

Clinical evidence and limitations: Early randomized controlled trials and small pilot studies in older adults and MCI patients report modest cognitive improvements and decreased peripheral inflammatory/oxidative markers with multi-strain probiotics or synbiotics [51,61]. However, heterogeneity in strains, dose, duration, and patient selection limits conclusions. Large, well-powered, strain-specific RCTs with mechanistic readouts (metabolomics, mitochondrial function, CSF biomarkers) are urgently needed. Representative human clinical trials investigating probiotic and prebiotic interventions in AD or mild cognitive impairment are summarized in Table 4.

Collectively, current clinical evidence suggests that microbiota-based interventions confer modest but reproducible benefits in cognitive and biochemical outcomes, particularly in early-stage disease such as mild cognitive impairment. Among these, multi-strain probiotic formulations containing *Lactobacillus* and *Bifidobacterium* species demonstrate the most consistent effects on cognitive scores and systemic oxidative stress markers, although outcomes remain heterogeneous across studies. Notably, strain-specific interventions such as *Bifidobacterium breve A1* and *Lactobacillus plantarum C29* have shown promising results in improving memory and attention, highlighting the importance of targeted microbial selection. Synbiotic approaches appear especially advantageous due to their synergistic enhancement in short-chain fatty acid production and anti-inflammatory effects. However, the current body of evidence is limited by small sample sizes, short intervention durations, and variability in study design, preventing definitive conclusions regarding optimal formulations. Future large-scale, well-controlled trials incorporating mechanistic endpoints (e.g., metabolomics, mitochondrial function, and neuroimaging biomarkers) are essential to establish efficacy and guide precision microbiome-based therapies in Alzheimer’s disease.

### 6.2. Dietary Modulation—Polyphenols, Mediterranean Diet, Stingless Bee Honey, Fibre

Rationale: Diet is the principal and most tractable modifier of the microbiome. Diets rich in fibre and polyphenols increase SCFA-producing taxa and antioxidant capacity, while Western diets promote dysbiosis, endotoxemia, and metabolic inflammation that exacerbate oxidative stress and mitochondrial dysfunction [34].

Evidence: Epidemiological studies link Mediterranean-type diets with lower AD risk and improved cognitive trajectories, effects partially mediated by beneficial shifts in gut microbial composition and metabolite profiles (polyphenol metabolites, increased butyrate) [64]. Preclinical and small clinical studies demonstrate that dietary polyphenols (e.g., from berries, green tea) and high-fibre intake reduce oxidative markers, support mitochondrial resilience, and attenuate Aβ pathology [16].

Stingless bee honey (SBH): Natural products with antioxidant and prebiotic properties such as stingless bee honey, modulate gut microbiota and possess free-radical scavenging activity. Experimental AD models show SBH supplementation can reduce oxidative stress and improve behavioural outcomes, suggesting a dual microbiome-mediated and direct antioxidant mechanism [62,63].

Practical considerations: Dietary interventions are safe and scalable but require long-term adherence and individualized tailoring to baseline microbiota and metabolic status. Combining diet with targeted pre/probiotics may yield synergistic benefit.

Plant-derived products and phytochemicals: In addition to dietary patterns, specific plant products and phytochemicals have been shown to modulate gut microbiota composition and function, enhancing SCFA production and antioxidant capacity while reducing pro-inflammatory taxa [81]. Examples include polyphenol-rich extracts from berries, green tea catechins, curcumin, and flavonoid-containing foods, which can promote beneficial genera such as *Bifidobacterium* and *Faecalibacterium*, restore intestinal barrier integrity, and mitigate systemic inflammation. These microbiota-mediated effects may contribute to reduced oxidative stress, improved mitochondrial function, and attenuation of amyloid and tau pathology in AD models. Integrating plant-derived bioactive with other microbiota-targeted interventions (probiotics, prebiotics, dietary fibers) may yield additive or synergistic neuroprotective benefits.

### 6.3. Faecal Microbiota Transplantation (FMT)—Evidence, Risks, Ethics

Rationale and evidence: FMT transfers entire microbial communities and can produce rapid reconfiguration of host microbiomes. In animal models, FMT from healthy donors improves cognition and reduces amyloid/oxidative markers, whereas FMT from AD donors can transfer pathology [13,51], providing strong causal evidence that community-level microbiota changes modify AD-related phenotypes.

Clinical state and risks: Although faecal microbiota transplantation (FMT) is an established and guideline-supported therapy for recurrent *Clostridioides difficile* infection, its application in neurodegenerative disorders, including AD, remains highly experimental. Human clinical data in AD are extremely limited. Small, exploratory FMT studies in neuropsychiatric and neurodegenerative disorders show variable outcomes and raise safety concerns, including pathogen transmission and unintended immune activation. Ethical and regulatory considerations include rigorous donor screening, long-term monitoring, and the definition of appropriate clinical endpoints. Given these uncertainties, FMT for AD should currently be conducted only within controlled clinical trials with strict safety oversight [61].

### 6.4. Next-Generation Approaches—Engineered Probiotics, Postbiotics, Metabolite Mimetics

Rationale: Precision approaches aim to deliver defined functions (e.g., targeted SCFA release, antioxidant enzyme expression, amyloid-degrading activities) rather than whole organisms. Postbiotics (microbial metabolites or inactivated microbial components) and small-molecule metabolite mimetics can provide therapeutic molecules without the risks of live microbes. Engineered probiotics can be designed to sense inflammatory signals and respond by producing neuroprotective metabolites or enzymes.

Evidence and status: Preclinical studies demonstrate that genetically engineered strains producing butyrate or antioxidant peptides can protect neuronal cells, enhance mitochondrial biogenesis, and reduce Aβ aggregation [17,34,39]. Postbiotics such as defined SCFA formulations and purified indole derivatives show promise in rodent AD models by restoring redox balance and mitochondrial function [60]. Translation to humans requires attention to pharmacokinetics, CNS penetrance of metabolites, host-microbe interactions, and regulatory pathways.

Integration and combination therapies: Given the multifactorial nature of AD, combining microbiome modulation with standard care (e.g., lifestyle, vascular risk management) or emerging disease-modifying agents may produce additive or synergistic effects. Trials designed to test combination regimens with mechanistic biomarkers (oxidative stress markers, mitochondrial function assays, CSF Aβ/tau) will be most informative.

## 7. Biomarker and Translational Frontiers

The gut-brain axis provides a rich and largely untapped source of biomarkers for Alzheimer’s disease, spanning microbial metabolites, host-microbe metabolic signatures, and advanced computational tools. Table 5 summarizes emerging microbiota-derived biomarkers and translational strategies, highlighting their biological sources, mechanistic relevance to AD pathology, and potential clinical applications, which are discussed in detail in the following sections.

### 7.1. Microbiota-Derived Metabolites as Biomarkers

Short-chain fatty acids (SCFAs), bile acids, and tryptophan metabolites represent the most promising microbial metabolites linked to AD. Reduced levels of butyrate and propionate are consistently observed in patients with mild cognitive impairment and AD, reflecting gut dysbiosis and impaired neuroprotective signalling [47]. Conversely, elevated secondary bile acids such as deoxycholic acid correlate with cognitive decline and hippocampal atrophy, suggesting their neurotoxic role in amyloid and mitochondrial pathways [16]. Tryptophan catabolites, particularly kynurenine derivatives, modulate neuroinflammation and excitotoxicity; shifts in the kynurenine-to-tryptophan ratio are emerging as predictive markers of cognitive decline [82]. These metabolites not only reflect microbiota composition but also provide mechanistic insight into oxidative stress, mitochondrial dysfunction, and amyloid aggregation.

### 7.2. Multi-Omics Integration

Single-omics approaches are insufficient to capture the complexity of gut–brain crosstalk in AD. Multi-omics integration combining metagenomics, metabolomics, lipidomics, and proteomics enables a holistic systems-level view [83]. For example, linking microbial gene pathways with host plasma metabolomes has revealed distinct signatures of lipid dysregulation, systemic inflammation, and mitochondrial stress in AD patients, implicating gut microbes in broader metabolic and immune networks [84].

Importantly, emerging studies now integrate gut microbial profiles with peripheral blood biomarkers (e.g., inflammatory cytokines, plasma Aβ42/40 ratios, neurofilament light chain) and neuroimaging measures such as hippocampal atrophy on MRI or amyloid/tau PET burden. These combined datasets demonstrate correlations between specific microbial taxa, circulating inflammatory mediators, and structural or molecular brain changes, strengthening the mechanistic link between peripheral dysbiosis and central neurodegeneration [85,86].

Recent longitudinal multi-omics studies demonstrate that combined microbial, metabolic, inflammatory, and neuroimaging profiles may outperform conventional CSF Aβ/tau measures alone in predicting early cognitive decline [87]. This integrative strategy enables more precise disease stratification, improves early detection, and facilitates identification of multidimensional therapeutic targets.

### 7.3. Circulating vs. Faecal Biomarkers

Faecal microbiota composition and metabolites are direct readouts of gut dysbiosis, but their clinical utility is constrained by variability in diet, transit time, and sampling protocols [13]. Circulating biomarkers such as plasma SCFAs, bile acids, and microbial tryptophan metabolites offer more stable and clinically scalable measures of microbiome function [60]. For instance, serum indole-3-propionate, a microbiota-derived antioxidant metabolite, has been associated with preserved hippocampal volume and slower cognitive decline [80,88]. Moving forward, a dual approach integrating faecal and circulating measures will be required to capture both local gut dysbiosis and systemic metabolic consequences in AD.

### 7.4. AI and Machine Learning Predictive Models

The heterogeneity of AD necessitates advanced computational frameworks to extract predictive power from high-dimensional microbiome datasets. Artificial intelligence (AI) and machine learning (ML) models have successfully classified AD patients and controls based on gut microbiota profiles with >85% accuracy in recent studies [85]. Integration of multi-omics layers further enhances predictive capacity, enabling individualized risk stratification and monitoring of therapeutic responses [83,86]. Explainable AI (XAI) models are now being developed to identify key microbial taxa and metabolites driving predictions, providing mechanistic interpretability alongside diagnostic value [86]. Ultimately, AI-guided biomarker panels may transform microbiota research into clinically actionable diagnostics for AD. Together, these biomarker and computational advances position the gut microbiome not only as a mechanistic contributor to AD, but also as a practical platform for early diagnosis and precision intervention.

## 8. Challenges and Future Perspectives

### 8.1. Individual Variability: Genetics, Diet, Environment

Inter-individual heterogeneity in baseline microbiota composition, host genetics, diet, medication use, and environmental exposures complicates both biomarker discovery and therapeutic generalization [89]. Genotype–microbiome interactions (e.g., APOE status) can modify microbial effects on metabolism and immune responses, producing variable downstream impacts on oxidative stress and mitochondrial resilience. Dietary patterns and polypharmacy further shape microbial ecology, meaning that “one-size-fits-all” probiotic or dietary prescriptions are unlikely to be universally effective. Future trials must incorporate stratification by host genotype, baseline microbiome profiles, diet, and comorbidities, and use adaptive designs to identify responder subgroups [51,89].

### 8.2. Translational Barriers: From Animal Models to Human Disease

Preclinical models provide mechanistic and causal proof-of-concept, but species differences in microbiome composition, immune regulation, and brain physiology limit direct extrapolation [17]. Germ-free and antibiotic-treated rodent studies are invaluable for establishing causality, yet they do not recapitulate human microbial complexity, lifespan, or environmental diversity. Translational pipelines must therefore emphasize (1) humanized microbiome models (donor-derived FMT into gnotobiotic hosts), (2) large-scale longitudinal human cohorts with multi-omics, and (3) phase 0/early-phase clinical studies that include mechanistic readouts (metabolomics, mitochondrial function, CSF biomarkers) to bridge animal-to-human gaps [13,61].

### 8.3. Regulatory and Safety Issues: FMT, Engineered Microbes, Live Biotherapeutics

Interventions such as fecal microbiota transplantation (FMT), next-generation live biotherapeutics, and engineered probiotics raise regulatory and safety considerations that extend beyond conventional pharmaceuticals. Risks include inadvertent pathogen transfer, long-term ecological shifts, horizontal gene transfer, and unanticipated immune consequences [22]. Regulatory agencies are still developing frameworks for live microbial therapeutics and postbiotics; early engagement with regulators, standardized donor screening, stringent manufacturing (GMP) processes, and long-term safety monitoring are non-negotiable prerequisites for clinical development [86]. Ethical considerations especially for vulnerable populations (older adults with cognitive impairment) require particular care in informed consent and risk–benefit assessment.

### 8.4. Personalized Microbiome Medicine: Path to Prevention and Therapy

Despite these barriers, the heterogeneity of AD simultaneously creates an opportunity: precision microbiome medicine. Integrating host genomics, baseline microbiome signatures, metabolomic phenotypes, and lifestyle data can enable rational matching of interventions (e.g., specific probiotic strains, dietary patterns, or postbiotic formulations) to individuals most likely to benefit [22,89]. Adaptive clinical trial designs, *n*-of-1 trials, and AI-guided stratification will be central to this approach. Importantly, preventive strategies targeting midlife metabolic risk, diet, and microbiome resilience may yield greater public-health impact than late-stage disease treatment. Achieving this will require interdisciplinary consortia, standardized protocols, and investment in longitudinal, multi-ethnic cohorts.

### 8.5. Research Infrastructure and Standardization Needs

Progress will be accelerated by harmonizing sampling protocols, sequencing and metabolomics pipelines, and analytic standards. Open data sharing, consensus on minimal reporting standards, and cross-cohort validations are necessary to build robust, generalizable biomarker panels [22,60]. Equally important is investment in translational platforms (humanized models, organoids, ex vivo assays) that can test mechanisms and safety before large clinical trials.

### 8.6. Final Perspective

In sum, microbiome-targeted strategies for AD are scientifically promising and uniquely positioned to address converging drivers oxidative stress, mitochondrial dysfunction, and amyloid pathology but they will require precision approaches, stringent safety frameworks, and rigorous translational science. If these challenges are met, the microbiota-gut-brain axis could provide the missing systemic lever needed to shift AD care from symptomatic management to prevention and disease modification.

## 9. Conclusions

The cumulative evidence underscores that gut microbiota is not a peripheral player but a systemic driver of Alzheimer’s disease (AD) pathogenesis, orchestrating a cascade that spans oxidative stress, mitochondrial dysfunction, amyloid aggregation, and neuroinflammation. Dysbiosis-derived metabolites such as lipopolysaccharides, secondary bile acids, and tryptophan catabolites exacerbate redox imbalance and mitochondrial injury, while depletion of beneficial short-chain fatty acids weakens neuroprotective and anti-inflammatory defences [16,52]. This mechanistic convergence establishes the microbiome-gut-brain axis as a central pathogenic hub that accelerates amyloidogenic cascades and synaptic dysfunction in AD.

Beyond pathology, the microbiome represents a novel therapeutic frontier that transcends the traditional amyloid-tau paradigm. Interventions such as probiotics, prebiotics, dietary modulation, faecal microbiota transplantation, and next-generation microbial engineering show growing promise in preclinical and early clinical studies, with the capacity to restore antioxidant capacity, improve mitochondrial function, and mitigate amyloid pathology [39,51]. Yet, translation requires caution, as variability in host genetics, diet, environment, and baseline microbiota can profoundly influence outcomes.

Importantly, microbiota-based strategies are most applicable to sporadic AD, which constitutes most cases and is heavily influenced by modifiable systemic and environmental factors. While familial AD provides mechanistic insight into amyloid-driven neurodegeneration, microbiome modulation is unlikely to override deterministic genetic mutations but may still modify downstream inflammatory and oxidative cascades. Thus, the therapeutic promise of the microbiota-gut-brain axis lies primarily in prevention, risk modification, and early intervention in sporadic AD.

The future of AD therapeutics demands precision and integrative strategies that combine multi-omics biomarkers, artificial intelligence-based predictive modelling, and individualized microbiome modulation. Such approaches could enable early detection, risk stratification, and personalized prevention, reshaping AD management from late-stage intervention toward proactive, systems-level medicine [85,86].

In conclusion, targeting the gut microbiota holds transformative potential for altering the trajectory of AD. By integrating microbiome science with neurology, immunology, and systems biology, we may finally unlock disease-modifying interventions capable of addressing the complex interplay of oxidative, metabolic, and inflammatory drivers of neurodegeneration.

## Figures and Tables

**Table 1:** Gut Microbiota Alterations in Alzheimer’s Disease (AD) from Humans and Animals.

Study	Model (Human/Animal)	Microbial Change (↑/↓)	Associated Pathology (Cognition, Aβ, Inflammation)
[34]	Human, AD patients vs. controls	↓ *Faecalibacterium*, ↓ *Roseburia*, ↓ *Bifidobacterium*; ↑ *Escherichia/Shigella*, ↑ *Enterobacteriaceae*	Reduced SCFAs, ↑ systemic inflammation, ↑ oxidative stress
[44]	Human, metagenomics	↓ Butyrate-producing bacteria; ↑ *Ruminococcus gnavus*	↑ Pro-inflammatory cytokines, cognitive decline
[45,46]	Human cohort, plasma microbiome link	Dysbiosis → ↑ LPS load, ↓ antioxidant taxa	↑ Systemic inflammation, ↑ oxidative damage, worsened amyloid pathology
[47]	Human, metabolomic profiling	↓ Butyrate, ↓ propionate	Impaired mitochondrial function, reduced antioxidant defenses
[7]	Human, fecal microbiome linked with CSF biomarkers	Altered microbiota correlated with ↑ tau, ↓ Aβ42	Potential biomarker for AD progression
[43]	Human, post-mortem brain & plasma	↑ Gram-negative taxa derived LPS	LPS co-localized with amyloid plaques, ↑ microglial activation
[9]	Germ-free AD transgenic mice	Absence of microbiota → ↓ amyloid burden	GF mice show reduced Aβ deposition vs. colonized mice
[48]	APP/PS1 mice, antibiotic treatment	Depletion of gut microbiota	↓ Amyloid plaques, ↓ neuroinflammation, improved cognition
[49]	Faecal microbiota transplantation (FMT) from AD patients to GF mice	AD-FMT → ↑ dysbiotic taxa	↑ Amyloid load, mitochondrial dysfunction, impaired memory
[29]	Mouse AD model, metabolite analysis	↑ Secondary bile acids, ↑ tryptophan catabolites	Disrupted BBB, ↑ microglial activation, ↑ neurodegeneration

↑ and ↓ indicate relative increase or decrease compared with healthy controls or baseline conditions. AD, Alzheimer’s disease; Aβ, amyloid-β; BBB, blood–brain barrier; CSF, cerebrospinal fluid; FMT, faecal microbiota transplantation; GF, germ-free; LPS, lipopolysaccharide; SCFAs, short-chain fatty acids.

**Table 2:** Mechanistic Intersections of Gut Dysbiosis with Alzheimer’s Disease Pathways.

Pathway	Microbial Link	Mechanistic Outcome in AD	Representative Studies
Oxidative Stress	↓ SCFA-producing bacteria (*Faecalibacterium*, *Roseburia*) ↑ Gram-negative taxa (↑ LPS, TMAO producers)	Impaired Nrf2 antioxidant defense, ↑ ROS, lipid peroxidation, DNA damage → accelerates amyloidogenesis	[52,53,54]
Mitochondrial Dysfunction	↑ LPS and bacterial amyloids ↓ Indole derivatives & SCFAs	Mitochondrial permeability transition, ATP depletion, impaired oxidative phosphorylation, ↑ ROS and Aβ aggregation	[17,32,55]
Amyloid Pathology	↑ *Escherichia/Shigella*, ↑ bacterial amyloids (curli fibres) ↑ Secondary bile acids	Cross-seeding of bacterial amyloids with host Aβ, tau hyperphosphorylation, prion-like seeding, accelerated plaque formation	[31,56]
Neuroinflammation & BBB Integrity	↑ LPS, ↑ pro-inflammatory cytokines ↓ SCFA-producing bacteria	TLR4-mediated microglial activation, cytokine storm, BBB disruption (↓ tight junctions), vagus nerve dysregulation	[14,53,57]

↑ and ↓ indicate relative increase or decrease compared with healthy or non-diseased states. AD, Alzheimer’s disease; Aβ, amyloid-β; BBB, blood–brain barrier; LPS, lipopolysaccharide; ROS, reactive oxygen species; SCFAs, short-chain fatty acids; TMAO, trimethylamine-N-oxide.

**Table 3:** Microbiota-based therapeutic interventions in Alzheimer’s disease: mechanisms, evidence, and translational potential.

Intervention	Mechanisms of Action	Preclinical Evidence	Clinical Evidence	Advantages	Limitations/Challenges
Probiotics (*Lactobacillus rhamnosus*, *L. plantarum C29*, *Bifidobacterium breve A1*, *B. longum*, *multi-strain blends*)	↑ SCFAs (butyrate), ↓ ROS & LPS, ↑ Nrf2/GSH antioxidant defense, ↓ microglial activation, ↓ Aβ aggregation	Improved cognition, reduced hippocampal oxidative stress, ↓ amyloid burden in transgenic AD mouse models (APP/PS1) and rodent studies [45]	Small RCTs show modest cognitive improvement (MMSE) and reduced oxidative/inflammatory biomarkers; strain-dependent effects [49,61]	Non-invasive, safe, widely accessible	Strain-specific variability, inconsistent outcomes, lack of large-scale RCTs
Prebiotics (inulin, GOS, FOS, resistant starch, arabinoxylans)	Promote SCFA-producing taxa (e.g., *Faecalibacterium*), enhance gut barrier integrity, ↓ systemic inflammation and endotoxemia	Improved synaptic plasticity, reduced ROS, and tau pathology, enhanced mitochondrial function in AD models [8,58]	Limited human data; some improvements in metabolic and inflammatory markers, minimal cognitive endpoints	Can synergize with probiotics (synbiotics), dietary-based approach	Inter-individual microbiome variability, GI intolerance, limited cognitive evidence
Dietary Modulation (Mediterranean diet, polyphenols, stingless bee honey, high-fiber diets)	↑ Antioxidant capacity, ↑ SCFAs, ↓ pro-inflammatory taxa, polyphenol metabolites cross BBB, SBH acts as antioxidant & prebiotic	Mediterranean diet & polyphenols reduce oxidative stress, improve mitochondrial function, ↓ Aβ in AD models [48,62]	Epidemiological evidence of reduced AD risk; small clinical trials show biomarker and cognitive improvements [16,63,64]	Safe, long-term, culturally adaptable, scalable	Requires long-term adherence, individualized response, trial durations are long
Fecal Microbiota Transplantation (FMT)	Rapid microbiome reconstitution, ↑ SCFA production, ↓ neuroinflammation, ↓ Aβ/tau pathology	FMT from healthy donors improves cognition and reduces amyloid/oxidative burden; AD donor FMT transfers pathology [13,49]	Very limited exploratory data in neurodegeneration, high safety concerns [61]	Strong causal evidence from preclinical studies, system-wide effects	Risk of pathogen transfer, immune reactions, ethical and regulatory barriers, still experimental
Next-Generation Approaches (engineered probiotics, postbiotics such as butyrate, indole derivatives, microbial metabolites)	Targeted delivery of neuroprotective metabolites, modulation of mitochondrial function, antioxidant signaling, amyloid degradation	Engineered strains and postbiotics improve neuronal survival, ↓ oxidative stress, restore mitochondrial bioenergetics [17,45,60]	Limited or no AD-specific clinical trials; early-phase studies ongoing	Precision-based, safer than live microbes, scalable as therapeutics	Translational barriers (bioavailability, CNS penetration, pharmacokinetics)

↑ and ↓ indicate relative increase or decrease compared with baseline or untreated conditions. AD, Alzheimer’s disease; Aβ, amyloid-β; BBB, blood–brain barrier; FMT, fecal microbiota transplantation; GOS, galacto-oligosaccharides; FOS, fructo-oligosaccharides; GI, gastrointestinal; LPS, lipopolysaccharide; ROS, reactive oxygen species; RCTs, randomized controlled trials; SBH, stingless bee honey; SCFAs, short-chain fatty acids.

**Table 4:** Clinical trials investigating probiotic and prebiotic interventions in Alzheimer’s disease and mild cognitive impairment.

Intervention (Probiotic/Prebiotic)	Study Model/Patients	Study Duration	Target Effects	Proposed Mechanisms	Reference
Multispecies probiotic (*Lactobacillus* + *Bifidobacterium* strains)	60 AD patients (RCT)	12 weeks	Improved MMSE scores, reduced malondialdehyde and hs-CRP	Anti-inflammatory effects, improved antioxidant capacity	[68,69]
Probiotic mixture (*Lactobacillus acidophilus*, *Bifidobacterium bifidum*, *L. fermentum*, *L. casei*)	52 AD patients	12 weeks	Improved cognitive function and metabolic profiles	Reduced oxidative stress, modulation of gut microbiota	[68,70,71]
Synbiotic formulation (probiotic + prebiotic fiber)	Mild cognitive impairment patients	12 weeks	Improved memory performance and metabolic biomarkers	Increased SCFA production and reduced systemic inflammation	[72,73]
*Bifidobacterium breve A1*	Older adults with MCI	16 weeks	Improved episodic memory and cognitive scores	Gut–brain axis modulation, anti-inflammatory activity	[74,75,76]
*Lactobacillus plantarum C29*	Elderly individuals with MCI	12 weeks	Improved cognitive performance and attention	Reduced neuroinflammation, improved gut barrier function	[77,78,79]
Probiotic-fermented milk (*Lactobacillus* + *Bifidobacterium*)	AD patients	12 weeks	Improved cognitive scores and insulin metabolism	Gut microbiota modulation, metabolic regulation	[68,78]
Prebiotic supplementation (inulin/FOS-based)	Older adults/MCI	12–24 weeks	Improved metabolic and inflammatory markers (limited cognitive improvement)	SCFA-mediated anti-inflammatory effects	[12]
Synbiotic (multi-strain + FOS/inulin)	Elderly subjects with cognitive decline	12 weeks	Improved cognitive performance and gut microbiota diversity	Synergistic microbiome modulation	[73,80]

AD, Alzheimer’s disease; MCI, mild cognitive impairment; MMSE, Mini-Mental State Examination; SCFAs, short-chain fatty acids; hs-CRP, high-sensitivity C-reactive protein; RCT, randomized controlled trial.

**Table 5:** Emerging microbiota-derived biomarkers and translational tools in Alzheimer’s disease.

Biomarker/Modality	Biological Source	Mechanistic Link to AD	Translational Potential	Representative Studies
Short-chain fatty acids (SCFAs: butyrate, propionate)	Faecal, plasma	↓ Butyrate/propionate → impaired Nrf2 antioxidant signalling, ↑ ROS, reduced synaptic resilience	Early diagnostic marker; therapeutic monitoring for dietary/probiotic interventions	[47]
Bile acids (secondary BAs: deoxycholic acid, lithocholic acid)	Plasma, CSF	↑ Secondary bile acids → mitochondrial stress, Aβ aggregation, hippocampal atrophy	Plasma BA ratios as non-invasive metabolic markers; adjunct to CSF Aβ/tau	[16]
Tryptophan catabolites (kynurenine/indole pathway)	Plasma, CSF	↑ Kynurenine: tryptophan ratio → excitotoxicity, neuroinflammation; ↓ indole-3-propionate → loss of antioxidant defense	Predictive marker of cognitive decline; stratification for immuno-metabolic interventions	[80,82]
Multi-omics integration (metagenomics + metabolomics + lipidomics + proteomics)	Faecal, plasma, CSF	Dysbiotic microbial gene pathways linked to host lipid metabolism and mitochondrial dysfunction	Outperforms CSF Aβ/tau in early detection; systems-level biomarker panels	[83,84]
Circulating vs. faecal biomarkers (dual approach)	Faecal (composition, SCFAs, BAs); plasma (SCFAs, tryptophan metabolites)	Faecal: local dysbiosis; Circulating: systemic metabolic consequences	Combined profiles capture gut and systemic pathology; clinically scalable	[13,60]
AI & machine learning classifiers (microbiome-based)	High-dimensional microbiota and multi-omics datasets	Identification of key microbial taxa/metabolites driving AD pathology	>85% accuracy in classifying AD vs. controls; XAI models provide interpretability	[85,86]

↑ and ↓ denote relative increases or decreases compared with cognitively normal controls or baseline conditions. AD, Alzheimer’s disease; Aβ, amyloid-β; BA(s), bile acid(s); CSF, cerebrospinal fluid; ROS, reactive oxygen species; SCFAs, short-chain fatty acids; XAI, explainable artificial intelligence.

## Data Availability

No new data were created or analyzed in this study. Data sharing is not applicable to this article.

## Supplementary Materials

The following supporting information can be downloaded at: https://www.mdpi.com/article/10.3390/biomedicines14040860/s1, PRISMA 2020 checklist.

## Abbreviations

The following abbreviations are used in this manuscript:

AD	Alzheimer’s Disease
AI	Artificial Intelligence
APC	Article Processing Charge
APOE	Apolipoprotein E
APP	Amyloid Precursor Protein
ARIA	Amyloid-Related Imaging Abnormalities
ATP	Adenosine Triphosphate
BA	Bile Acids
BBB	Blood-Brain Barrier
CNS	Central Nervous System
CSF	Cerebrospinal Fluid
DNA	Deoxyribonucleic Acid
FMT	Fecal Microbiota Transplantation
FOS	Fructooligosaccharides
GF	Germ-Free
GI	Gastrointestinal
GPT	Generative Pre-trained Transformer
GSH	Glutathione
LPS	Lipopolysaccharide
MCI	Mild Cognitive Impairment
ML	Machine Learning
NADPH	Nicotinamide Adenine Dinucleotide Phosphate (Reduced Form)
RAGE	Receptor for Advanced Glycation End-products
ROS	Reactive Oxygen Species
SBH	Stingless Bee Honey
SCFA	Short-Chain Fatty Acids
TCA	Tricarboxylic Acid Cycle
TMAO	Trimethylamine N-oxide
URL	Uniform Resource Locator
XAI	Explainable Artificial Intelligence
